# Association between Plasma N-6 Polyunsaturated Fatty Acids Levels and the Risk of Cardiovascular Disease in a Community-based Cohort Study

**DOI:** 10.1038/s41598-019-55686-7

**Published:** 2019-12-17

**Authors:** Wei-Sin Yang, Yun-Yu Chen, Pei-Chun Chen, Hsiu-Ching Hsu, Ta-Chen Su, Hung-Ju Lin, Ming-Fong Chen, Yuan-Teh Lee, Kuo-Liong Chien

**Affiliations:** 1 Institute of Epidemiology and Preventive Medicine, College of Public Health, National Taiwan University, Taipei, Taiwan; 2 Division of Cardiology, Department of Medicine, Taipei Veterans General Hospital, Taipei, Taiwan; 3 Department of Public Health, China Medical University, Taichung, Taiwan; 4 Department of Internal Medicine, National Taiwan University Hospital, Taipei, Taiwan; 5 Cardiovascular Research Laboratory, Cardiovascular Center, Clinical Outcome Research and Training Center, Big Data Center, China Medical University Hospital, China Medical University, Taichung, Taiwan

**Keywords:** Prognostic markers, Risk factors

## Abstract

Most studies support that saturated fatty acid replacement with polyunsaturated fatty acids (PUFAs) may reduce the risk of cardiovascular diseases (CVDs) and put emphasis on the effects of N-3 PUFAs. The reported relationships between N-6 PUFAs and CVD risks vary. We aimed to examine the associations between N-6 PUFA concentrations and CVD risks. In this community-based prospective cohort study on CVD-free patients at baseline (N = 1835, age: 60.6 ± 10.5 years, women: 44.5%), we measured the fatty acid concentrations in the blood using gas chromatography. Four hundred twenty-four participants developed CVDs during follow up. The total N-6 PUFA concentration was inversely associated with the CVD risk, with a 48% lower risk in the highest N-6 PUFA concentration quartile (hazard ratio = 0.52; *P* for trend <0.001). The estimated population attributable risk of N-6 PUFAs indicated that approximately 20.7% of CVD events would have been prevented if the plasma N-6 PUFA concentration had been higher than the median value. The total N-6 PUFA concentration presented the highest net reclassification improvement (NRI = 7.2%, *P* = 0.03) for predicting incident CVD. Further studies on N-6 PUFAs, diet habits, and their relationships with healthcare are warranted.

## Introduction

Atherosclerosis is a dynamic inflammatory process and one of the major underlying causes of cardiovascular diseases (CVDs), which may lead to great economic burden owing to its complications and related medical care^1^. From a clinical perspective, determining how to reduce inflammation-associated atherosclerosis may help target therapeutics beyond medications and improve daily clinical practice. The effects of dietary fat on atherosclerosis may be influenced by the inflow of lipids and lipoproteins, such as low-density lipoprotein (LDL) and high-density lipoprotein (HDL), from the plasma to the arterial wall^2^. Many normal metabolic processes cannot be carried out without a supply of essential fatty acids and metabolic enzymes. If essential fatty acids and metabolic enzymes are lacking, the synthesis of eicosanoids would be compromised^3–6^.

Most studies support the idea that saturated fatty acid (SFA) replacement with polyunsaturated fatty acids (PUFAs) may reduce the CVD risk^7,8^. Further, prior studies have demonstrated the plasma cholesterol-lowering effect of PUFAs in the human diet^9–11^. PUFAs are a family of lipids, including some subgroups (e.g. N-3 PUFAs [first double bond at carbon number 3] and N-6 PUFAs [first double bond at carbon number 6])^12^. For example, alpha-linolenic acid (ALA, C18:3 n-3), eicosapentaenoic acid (EPA, C20:5 n-3), and docosahexaenoic acid (DHA, C22:2 n-3) belong to the N-3 PUFA family. Conversely, linoleic acid (LA, C18:2 n-6), gamma-linolenic acid (GLA, C18:3 n6), and arachidonic acid (AA, C20:4 n-6) belong to the N-6 PUFA family^3,5^. In addition, delta-5 desaturase (D5D) and delta-6 desaturase (D6D) are the key enzymes involved in the metabolism of both N-3 and N-6 PUFAs, allowing the formation of long-chain metabolites^13,14^. Alterations in D5D and D6D activity may cause inflammation-associated diseases, such as type 2 diabetes mellitus (DM)^15^ and CVD^16–20^.

Prior studies put emphasis on the effects of N-3 PUFA consumption and have shown that N-3 PUFAs were correlated with the incident risk of coronary heart diseases (CHDs)^12^ and that N-6 and N-3 PUFAs have competing roles in the synthesis of eicosanoids^21^. Thus, balance intake of N-6 and N-3 PUFAs is necessary to maintain good health. In 2009, the American Heart Association recommended the consumption of at least 5–10% of energy from N-6 PUFAs^22^. Till date, the results regarding the relationship between N-6 PUFAs and CVD risks have been inconsistent. Moreover, related data in Asian populations are limited^23–29^. In this study, we aimed to examine the associations between higher concentrations of N-6 PUFAs and CVD risks.

## Results

**Figure 1:**
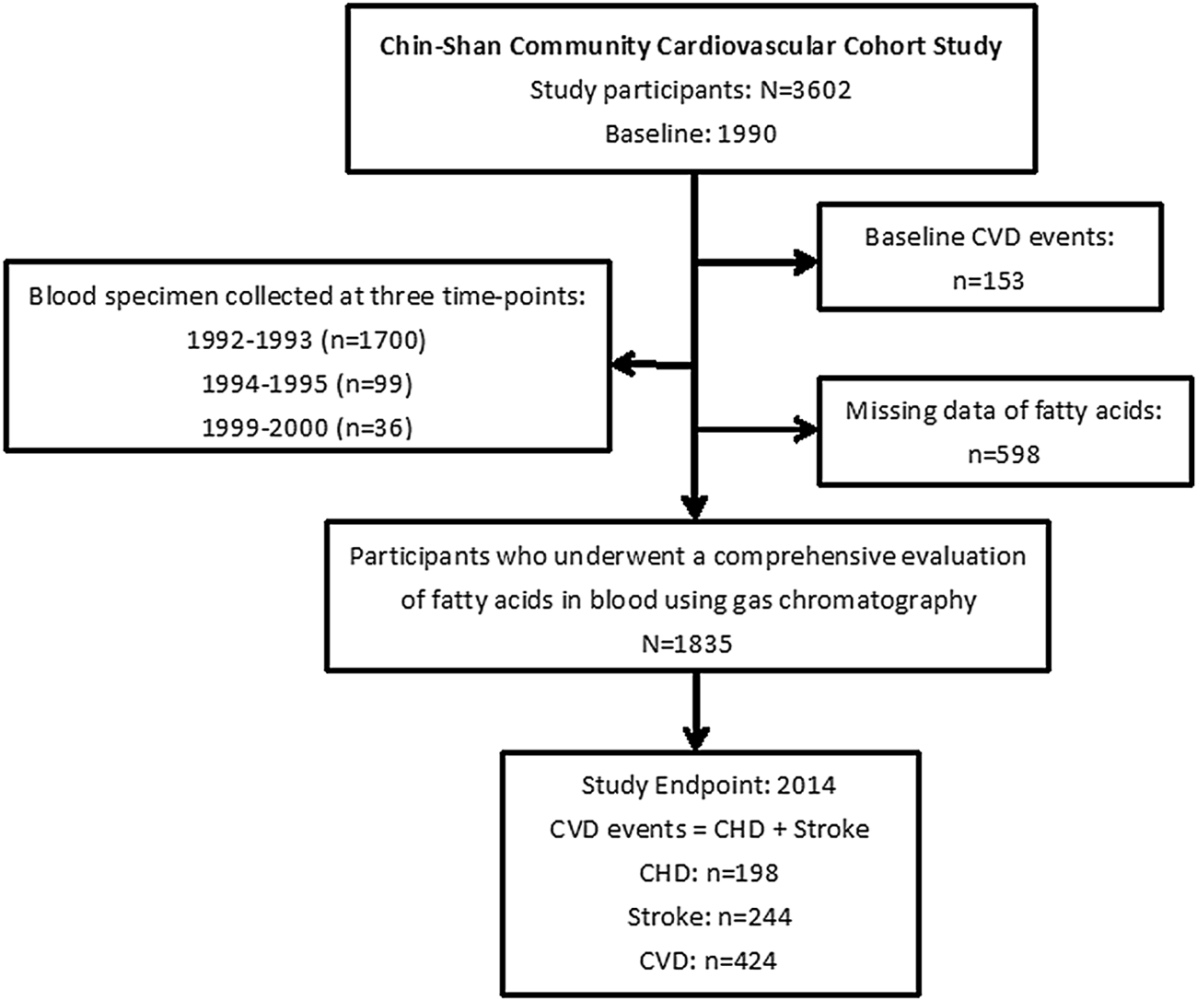
Flow chart of this study. CVD: cardiovascular diseases; CHD: coronary heart diseases.

A total of 1,703 men and 1,899 women living in Chin-Shan Township, Taiwan, were enrolled under regular follow-up, all of whom were free from CVD at baseline (Fig. 1)^30^. Table 1 shows the basic characteristics of the participants according to the LA quartiles at baseline. The participants in the upper quartiles were more likely to be younger and have lower systolic blood pressure, diastolic blood pressure, body mass index, triglyceride concentration, LDL cholesterol concentration, fasting glucose concentration, and higher HDL cholesterol concentration than those in the lowest quartile. In terms of plasma fatty acids, the participants in the upper quartiles had lower concentrations of saturated fat, trans fat, D6D, and higher concentrations of N-6 PUFAs (LA, GLA, and AA), N-3 PUFAs (ALA, EPA, and DHA), and D5D than those in the lowest quartiles. Regarding the categorical variables, the participants in the upper LA quartiles were less likely to smoke and consume alcohol, and a smaller percentage was likely to have a history of hypertension or type 2 DM.

**Table 1:** Basic characteristics of the participants according to the quartiles of linoleic acid (LA).

LA (% of total FAs)	Q1 (n = 456)	Q2 (n = 459)	Q3 (n = 460)	Q4 (n = 460)	P-value
	Mean SD	Mean SD	Mean SD	Mean SD	
Age, years	63.4 ± 10.1	61.6 ± 9.9	60.3 ± 10.8	57.1 ± 10.4	<0.001
Systolic BP, mmHg	132.1 ± 21.3	131.5 ± 22.0	129.1 ± 20.9	125.0 ± 20.5	<0.001
Diastolic BP, mmHg	79.1 ± 11.3	78.1 ± 11.4	78.4 ± 11.4	76.1 ± 10.2	<0.001
Body mass index, kg/m^2^	23.6 ± 3.6	23.5 ± 3.5	23.2 ± 3.1	22.9 ± 3.0	0.02
Triglyceride, mg/dL	148.5 ± 112.3	137.0 ± 101.3	129.3 ± 97.7	107.9 ± 69.5	<0.001
Total cholesterol, mg/dL	205.9 ± 47.3	205.4 ± 48.5	202.5 ± 43.3	199.2 ± 44.4	0.11
HDL cholesterol, mg/dL	45.8 ± 12.8	46.2 ± 12.2	46.9 ± 13.2	49.8 ± 13.8	<0.001
LDL cholesterol, mg/dL	146.4 ± 46.4	147.0 ± 47.1	142.2 ± 41.9	135.8 ± 42.6	<0.001
Fasting glucose, mg/dL	114.8 ± 35.4	116.7 ± 42.9	111.0 ± 35.8	108.7 ± 24.9	<0.001
CRP, mg/dL	0.3 ± 0.5	0.3 ± 0.5	0.2 ± 0.4	0.3 ± 0.6	0.47
Plasma fatty acids component^a^					
Saturated fat, % total FAs	55.2 ± 3.6	52.7 ± 2.4	50.1 ± 2.2	45.8 ± 3.1	<0.001
Polyunsaturated fat, % total FAs	24.3 ± 3.4	28.0 ± 2.3	30.6 ± 2.2	35.4 ± 3.2	<0.001
N-6 FAs,% total FAs	21.1 ± 3.0	24.5 ± 2.0	27.0 ± 1.9	31.4 ± 2.7	<0.001
LA	10.4 ± 1.7	13.7 ± 0.7	16.7 ± 1.0	21.5 ± 2.3	<0.001
GLA	0.21 ± 0.10	0.21 ± 0.09	0.23 ± 0.10	0.22 ± 0.10	<0.001
AA	2.5 ± 0.7	2.8 ± 0.8	3.2 ± 0.9	3.7 ± 1.1	<0.001
N-3 FAs, % total FAs	1.9 ± 0.8	2.1 ± 0.7	2.2 ± 0.7	2.5 ± 0.8	<0.001
ALA	0.4 ± 0.1	0.4 ± 0.1	0.5 ± 0.2	0.6 ± 0.2	<0.001
EPA	0.3 ± 0.2	0.4 ± 0.2	0.4 ± 0.2	0.4 ± 0.2	<0.001
DHA	1.5 ± 0.6	1.7 ± 0.6	1.8 ± 0.6	2.1 ± 0.7	<0.001
Trans fat	11.3 ± 3.0	9.4 ± 1.0	8.4 ± 1.0	6.9 ± 1.1	<0.001
D5D^a^	3.4 ± 1.0	3.9 ± 1.0	4.1 ± 1.2	4.5 ± 1.2	<0.001
D6D^a^	0.022 ± 0.03	0.015 ± 0.01	0.014 ± 0.01	0.01 ± 0.01	<0.001
Gender					0.17
Men, n (%)	266 (57.6)	259 (56.8)	260 (56.8)	234 (51.1)	
Women, n (%)	196 (42.4)	197 (43.2)	198 (43.2)	224 (48.9)	
Current smoker	231 (50.0)	193 (42.3)	203 (44.3)	172 (37.6)	<0.001
Alcohol intake	168 (36.4)	160 (35.1)	139 (30.4)	128 (28.0)	0.02
Marital status					0.03
Unmarried	23 (5.0)	19 (4.2)	21 (4.6)	11 (2.4)	
Married	355 (77.2)	378 (83.3)	376 (82.3)	391 (85.8)	
Separated	82 (17.8)	57 (12.6)	60 (13.1)	54 (11.8)	
Education years					0.25
<=9 years	449 (97.2)	432 (94.7)	435 (95.0)	439 (95.9)	
>9 years	13 (2.8)	24 (5.3)	23 (5.0)	19 (4.2)	
Job					<0.001
No or housewife	300 (64.9)	257 (56.4)	253 (55.2)	234 (51.1)	
Manual labor	131 (28.4)	134 (29.4)	151 (33.0)	180 (39.3)	
Official work	31 (6.7)	65 (14.3)	54 (11.8)	44 (9.6)	
Frequent exercise, yes	94 (20.4)	86 (18.9)	70 (15.3)	67 (14.6)	0.06
Family history of CAD, yes	34 (7.4)	47 (10.3)	35 (7.6)	39 (8.5)	0.37
Hypertension history, yes	196 (42.4)	189 (41.5)	170 (37.1)	127 (27.9)	<0.001
Type 2 diabetes mellitus, yes	87 (18.8)	90 (19.8)	65 (14.3)	55 (12.3)	<0.001

^a^Values represent relative weight % of fatty acids, except for D5D and D6D.

AA: arachidonic acid; ALA: alpha linolenic acid; BP: blood pressure; CAD: coronary artery diseases; CRP:C-reactive protein; D5D: delta-5 desaturase; D6D: delta-6 desaturase; DHA: docosahexaenoic acid; EPA: eicosapentaenoic acid; FAs: fatty acids; GLA: gamma-linolenic acid; HDL: high-density lipoprotein; LA: linoleic acid; LDL: low-density lipoprotein; PUFAs: polyunsaturated fatty acids.

A total of 424 individuals experienced CVD events during the follow-up period (a median of 15.9 years). After adjusting for multiple factors, the hazard ratio (HR) for the incident CVD risk in the highest quartile of the total N-6 PUFA concentration was 0.52 as compared with that in the lowest quartile (95% confidence interval [CI] = 0.38–0.71; *P* for trend <0.001; Table 2), and that for LA was 0.56 (95% CI = 0.40–0.77; *P* for trend < 0.001; Table 2). Conversely, N-3 PUFAs showed no significant protective effects in our data (total N-6 PUFAs in the highest quartile: HR = 0.81, 95% CI = 0.60–1.09; *P* for trend = 0.12; EPA in the highest quartile: HR = 0.98, 95% CI = 0.74–1.3; *P* for trend = 0.87; DHA in the highest quartile: HR = 1.04, 95% CI = 0.77–1.41; *P* for trend = 0.64; see Supplementary Table S2).

**Table 2:** Hazard ratio of incidence CVD according to N-6 PUFAs.

N-6 PUFAs	Q1	Q2	95% CI	Q3	95% CI	Q4	95% CI	P-trend
Median	20.99	24.61		27.15		31.22		
Case (n)	136	112		95		81		
Person-years	6466.5	6687.1		7453.8		8277.6		
Rates/1000 py	21	16.7		12.7		9.8		
Model 1	1	0.83	(0.64, 1.07)	0.66	(0.51, 0.87)	0.52	(0.39, 0.69)	<0.001
Model 2	1	0.83	(0.64, 1.08)	0.68	(0.52, 0.89)	0.51	(0.38, 0.68)	<0.001
Model 3	1	0.87	(0.67, 1.13)	0.71	(0.54, 0.94)	0.52	(0.38, 0.71)	<0.001
**LA**	**Q1**	**Q2**	**95% CI**	**Q3**	**95% CI**	**Q4**	**95% CI**	**p-trend**
Median	10.83	13.77		16.62		20.98		
Case (n)	121	121		107		75		
Person-years	6509.1	6666.7		7476.3		8295.5		
Rates/1000 py	18.6	18.2		14.3		9.0		
Model 1	1	1.08	(0.83, 1.40)	0.86	(0.66, 1.13)	0.56	(0.41, 0.77)	<0.001
Model 2	1	1.08	(0.83, 1.40)	0.86	(0.66, 1.13)	0.57	(0.42, 0.78)	<0.001
Model 3	1	1.05	(0.80, 1.37)	0.87	(0.67, 1.15)	0.56	(0.40, 0.77)	<0.001
**AA**	**Q1**	**Q2**	**95% CI**	**Q3**	**95% CI**	**Q4**	**95% CI**	**p-trend**
Median	2.02	2.62		3.22		4.21		
Case (n)	124	118		91		91		
Person-years	6607.2	7039.1		7471		7830.4		
Rates/1000 py	18.8	16.8		12.2		11.6		
Model 1	1	0.91	(0.70, 1.17)	0.69	(0.52, 0.91)	0.70	(0.52, 0.92)	0.004
Model 2	1	0.94	(0.73, 1.23)	0.68	(0.52, 0.91)	0.70	(0.53, 0.94)	0.005
Model 3	1	0.95	(0.73, 1.24)	0.72	(0.53, 0.96)	0.79	(0.58, 1.07)	0.06
**GLA**	**Q1**	**Q2**	**95% CI**	**Q3**	**95% CI**	**Q4**	**95% CI**	**p-trend**
Median	0.12	0.17		0.23		0.32		
Case (n)	107	100		120		97		
Person-years	7085.4	7198.9		7087.6		7575.7		
Rates/1000 py	15.1	13.9		16.9		12.8		
Model 1	1	0.99	(0.75, 1.32)	1.23	(0.94, 1.61)	0.95	(0.71, 1.26)	0.94
Model 2	1	0.98	(0.74, 1.31)	1.17	(0.89, 1.53)	0.89	(0.66, 1.19)	0.55
Model 3	1	0.99	(0.74, 1.32)	1.19	(0.90, 1.58)	0.93	(0.69, 1.25)	0.81

Model 1: age, gender.

Model 2: Model 1+ body mass index, smoking, alcohol consumption habits, marital status, education level, occupation, and regular exercise.

Model 3: Model 2+ baseline hypertension, diabetes, continuous high-density lipoprotein and low-density lipoprotein cholesterol values.

AA: arachidonic acid; CI: confidence interval; CVD: cardiovascular diseases; GLA: gamma-linolenic acid; LA: linoleic acid; PUFAs: polyunsaturated fatty acids; py: person-years.

Regarding the fatty acid metabolic enzymes, D5D was inversely associated with incident CVD, with a 33% lower risk in Model 2 (adjusted HR in the highest quartile = 0.67, 95% CI = 0.50–0.91; *P* for trend = 0.02; Table 3) among the participants in the highest quartile compared with those in the lowest quartile. However, the significant protective effect was attenuated after adjustment for additional clinical variables in Model 3. Regarding the P/S ratio, the HR in the highest quartile compared with that in the lowest quartile was 0.58 (95% CI = 0.42–0.80; *P* for trend < 0.001; see Supplementary Table S3).

**Table 3:** Hazard ratio of incidence CVD according to fatty acids metabolic enzymes D5D and D6D.

D5D	Q1	Q2	95% CI	Q3	95% CI	Q4	95% CI	P-trend
Median	2.68	3.50		4.14		5.37		
Case (n)	117	110		117		79		
Person-years	6706.3	7080.1		7214.6		7748.9		
Rates/1000 py	17.4	15.5		16.2		10.2		
Model 1	1	0.85	(0.65, 1.11)	0.91	(0.69, 1.18)	0.64	(0.48, 0.86)	0.01
Model 2	1	0.88	(0.67, 1.16)	0.95	(0.72, 1.25)	0.67	(0.50, 0.91)	0.02
Model 3	1	0.90	(0.68, 1.18)	1.02	(0.77, 1.35)	0.77	(0.56, 1.06)	0.16
**D6D**	**Q1**	**Q2**	**95% CI**	**Q3**	**95% CI**	**Q4**	**95% CI**	**p-trend**
Median	0.007	0.011		0.015		0.023		
Case (n)	97	98		118		110		
Person-years	7441.4	7312.8		6980.7		7015.1		
Rates/1000 py	13	13.4		16.9		15.7		
Model 1	1	1.06	(0.79, 1.42)	1.29	(0.97, 1.71)	1.24	(0.93, 1.65)	0.09
Model 2	1	0.97	(0.72, 1.30)	1.22	(0.92, 1.63)	1.14	(0.85, 1.52)	0.21
Model 3	1	0.93	(0.68, 1.26)	1.19	(0.88, 1.59)	1.16	(0.86, 1.55)	0.15

Model 1: age, gender.

Model 2: Model 1 + body mass index, smoking, alcohol consumption habits, marital status, education level, occupation, and regular exercise.

Model 3: Model 2 + baseline hypertension, diabetes, continuous high-density lipoprotein and low-density lipoprotein cholesterol values.

CI: confidence interval; CVD: cardiovascular diseases; D5D: delta-5 desaturase; D6D: delta-6 desaturase; PUFAs: polyunsaturated fatty acids; py: person-years.

For the trend of the CVD risk in the progressively increased N-6 PUFA quartile groups in both sexes, despite the significant interaction between N6-PUFAs and sexes, higher N-6 PUFA concentrations were found to be associated with lower CVD risks in both sex groups (all *P* for trend < 0.05, see Supplementary Table S4). The CVD risk in the female participants for Q3 (N-6 PUFA quartile) was significantly lower than that for Q1. However, we did not find a significantly lower CVD risk in the male participants for Q3 than for Q1 (see Supplementary Table S4).

As Table 1 shows an opposite proportion in the distribution of LA between the male and female participants, subgroup analyses for LA and N-6 PUFAs were performed via sex stratification after adjusting for multiple confounders. Higher LA quartiles were associated with lower incident CVD event risks in Model 0 (crude effect; Table 4) regardless of sex difference. However, we found a significant interaction between LA and sex (*P* = 0.003) and between the quartiles of LA and sex (*P* < 0.001) in Model 0. After adjusting for multiple confounders, the female participants achieved more significant benefits in the highest quartile of LA in reducing the incident CVD risk than did the male participants (HR = 0.57, 95% CI = 0.35–0.95; *P* for trend = 0.005; Table 4).

**Table 4:** Stratified effects of Linoleic acid (LA) by different genders in various models.

Gender	Models	Hazard ratio (95% confidence interval)				P trend
		Q1	Q2	Q3	Q4	
Men	Median (n)	10.8 (n = 261)	13.7 (n = 264)	16.6 (n = 260)	20.8 (n = 234)	
	Model 0	1 (reference)	0.89 (0.64, 1.25)	0.79 (0.56, 0.10)	0.57 (0.39, 0.83)	0.002
	Model 1	1 (reference)	0.96 (0.68, 1.34)	0.89 (0.63, 1.25)	0.74 (0.51, 1.08)	0.11
	Model 2	1 (reference)	0.93 (0.66, 1.31)	0.86 (0.61, 1.21)	0.75 (0.51, 1.11)	0.14
	Model 3	1 (reference)	0.86 (0.60, 1.22)	0.84 (0.59, 1.20)	0.71 (0.48, 1.06)	0.12
Women	Median (n)	10.8 (n = 195)	13.8 (n = 195)	16.6 (n = 200)	21.1 (n = 225)	
	Model 0	1 (reference)	1.24 (0.85, 1.82)	0.74 (0.48, 1.12)	0.42 (0.26, 0.66)	<0.001
	Model 1	1 (reference)	1.33 (0.91, 1.95)	0.82 (0.54, 1.24)	0.50 (0.32, 0.81)	<0.001
	Model 2	1 (reference)	1.37 (0.93, 2.03)	0.86 (0.56, 1.31)	0.53 (0.33, 0.85)	0.001
	Model 3	1 (reference)	1.43 (0.96, 2.13)	0.92 (0.60, 1.41)	0.57 (0.35, 0.95)	0.005

We tested if variables of gender and LA have statistical interaction effect, and found that a significantly statistical interaction of LA*gender (interaction P = 0.003) and quartiles of linoleic acid (LA)*gender (interaction P < 0.001) in Model 0.

Model 0: crude effect;

Model 1: age;

Model 2: Model 1+ body mass index, smoking, alcohol consumption habits, marital status, education level, occupation, and regular exercise;

Model 3: Model 2+ baseline hypertension, diabetes, continuous high-density lipoprotein and low-density lipoprotein cholesterol values.

LA showed significant protective effects in the two marker analyses in comparison with N-3 PUFAs and D5D (see Supplementary Table S5). The adjusted HRs for the highest LA and N-3 PUFA quartiles were 0.60 (95% CI = 0.38–0.93; *P* for trend = 0.02) and 1.02 (95% CI = 0.67–1.53; *P* for trend = 0.94), respectively; the HRs for the highest LA and D5D quartiles were 0.59 (95% CI = 0.38–0.92; *P* for trend = 0.02) and 0.98 (95% CI = 0.64–1.50; *P* for trend = 0.91), respectively.

**Figure 2:**
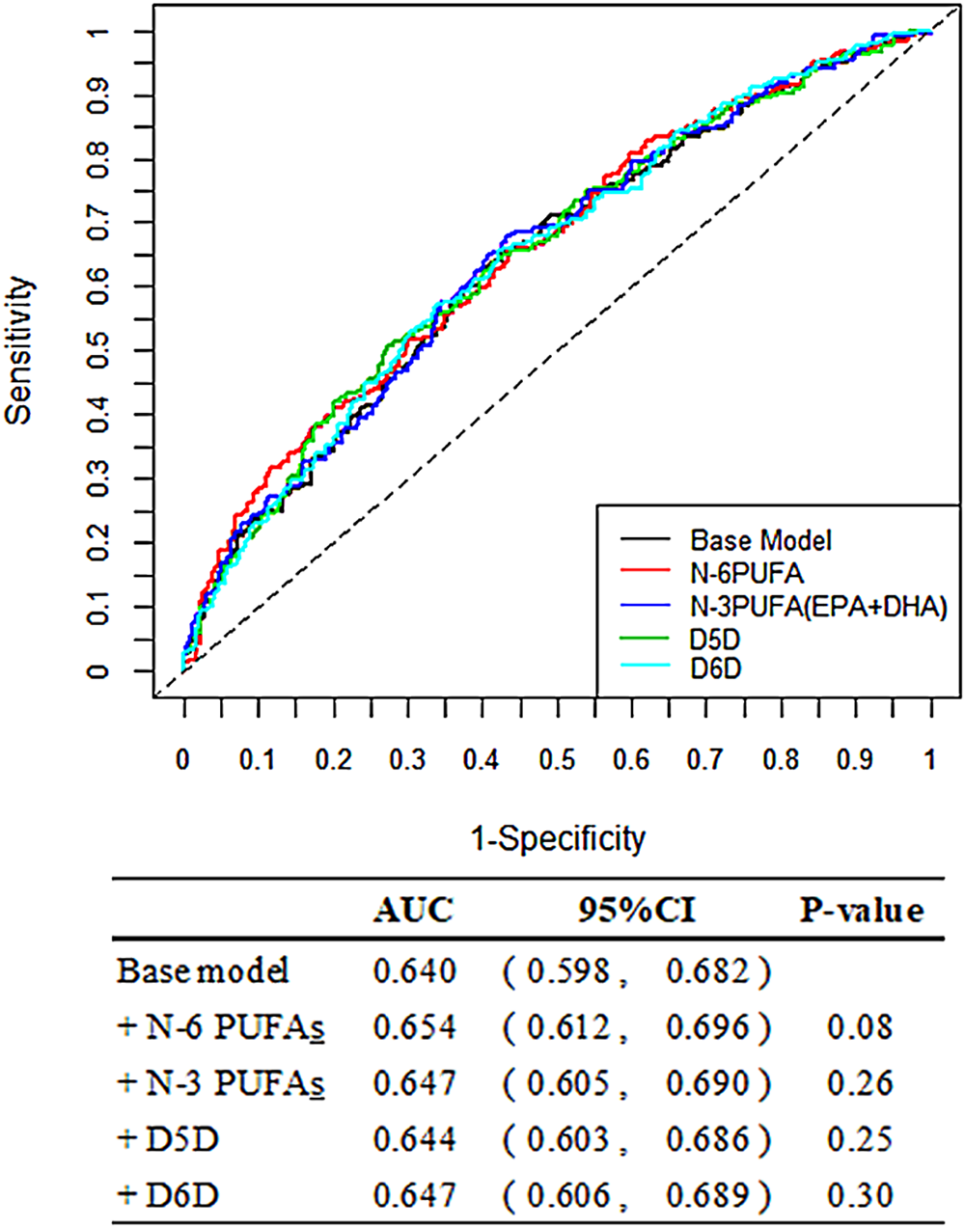
Area under the ROC curves of different fatty acids comparison with the base model. The base model included: age, gender, BMI, smoking, alcohol consumption habits, marital status, education level, occupation, regular exercise, baseline hypertension, diabetes, high-density lipoprotein and low-density lipoprotein cholesterol. AUC: area under the ROC curve; CI: confidence interval; D5D: delta-5 desaturase; D6D: delta-6 desaturase; PUFAs: polyunsaturated fatty acids; ROC: receiver operating characteristic curve.

In the receiver operating characteristic curve analysis, the area under the curve increased from 0.64 in the baseline model to 0.65 in the additional N-6 PUFA model (see Fig. 2 for the performance measurement values). The reclassification improvement with and without N-6 PUFAs is listed in Supplementary Table S6. N-6 PUFAs presented the highest NRI for predicting incident CVD than base model (NRI = 7.2%, *P* = 0.03), which indicated that only the inclusion of N-6 PUFAs could improve predictions related to CVD (see Supplementary Table S7).

The estimated population attributable risks (PARs) of the N-6 PUFA concentration indicated that approximately 20.7% of CVD events would have been prevented if the plasma N-6 PUFA concentration had been higher than the median value (26% of the total fatty acids) (see Supplementary Table S8).

## Discussion

We demonstrated several findings in this study: (1) The total N-6 PUFA concentration was inversely associated with the CVD risk when compared across the highest quartile to the lowest quartile. The inverse associations of N-6 PUFAs and LA with the CVD risks were found especially in the female participants; (2) the estimated PAR of N-6 PUFAs indicated that 20.7% of CVD events would have been prevented if the plasma N-6 PUFA concentration had been higher than the median value (26% of the total fatty acids); (3) we did not find a significant inverse relationship of N-3 PUFAs with incident CVD. In addition, among the metabolic enzymes, D5D reduced the risk of CVD after adjusting for age, body mass index, smoking, alcohol consumption habits, marital status, educational level, occupation, and regular exercise. However, the inverse effect was attenuated after adding additional factors of baseline hypertension, DM, continuous LDL cholesterol concentration, and HDL cholesterol concentration.

Previous studies put great emphasis on N-3 PUFAs, especially on marine PUFAs (EPA + DHA), and have observed a cardio-protective effect^31,32^. However, our study failed to find a significant association between the concentrations of N-3 PUFAs and CVD risk. Another study on marine PUFAs using the same community-based cohort found that the relative risk of CVD events in the highest quartile compared with that in the lowest quartile was 0.88 for EPA (*P* for trend = 0.54) and 1.12 for DHA (*P* for trend = 0.94)^30^. Despite the longer follow-up period in our study, we still were unable to detect significant protective effects of marine fatty acids. A meta-analysis of 14 randomised trials (involving 20,485 patients with prior CVD) investigated the role of EPA and DHA supplementation in the secondary prevention of CVD. The authors found insufficient evidence for a secondary protective effect of N-3 fatty acid supplements against overall cardiovascular events^33^.

The clinical value of N-6 PUFAs for total mortality and CVD risks remains controversial^21,34^. In the Framingham Heart Study, higher marine N-3 PUFA concentrations were related to reduced CVD, ischaemic stroke, and mortality risks. However, the authors could not find the same effect in N-6 PUFAs. A meta-analysis pooling 30 prospective cohort studies with nearly 70,000 participants showed that higher concentrations of LA were significantly associated with lower total CVD, cardiovascular mortality, and ischaemic stroke risks^35^, while the concentrations of AA were not associated with cardiovascular outcomes. A systematic review reported that N-3 and N-6 PUFAs have competing roles in the production of anti-inflammatory and inflammatory eicosanoids^21^. Some studies have suggested that the EPA:AA ratio was associated with CHD risk, but they could not find a consistent conclusion regarding the same^34^.

Several sources support the proposition that dietary habits may influence plasma fatty acids and health. A prior study analysing 16 cohorts indicated that higher intake of vegetable oils and vegetable foods but lower intake of hard fats and animal foods may reduce the all-cause mortality risk^36^. An ecological study revealed that the 50-year CHD mortality had a significant ecologic correlation with dietary patterns^37^, such as consumption of rich vegetable foods and starch; however, consumption of lower sweet products and animal foods in Mediterranean and Japanese cohorts was inversely correlated with a lower CHD risk. LA could be obtained mainly from vegetable oils (e.g. corn, sunflower, safflower, and soy), accounting for 85–90% of dietary N-6 PUFAs. In most western countries, emphasis is placed on providing adequate intake of essential nutrients. In clinical practice, although firm requirements have not been established for healthy adults, estimates have been derived from studies in infants and hospitalised patients receiving total parenteral nutrition. These suggested the sufficiency of an LA intake of approximately 0.5–2% of energy^22^. In this study, we demonstrated that the N-6 PUFA and LA concentrations were inversely associated with the CVD risk, and the significance can be found especially in women and the latter; conversely, the concentrations of N-3 PUFAs were not associated with decreased CVD risks in our cohort during the long-term follow-up period. We failed to explain the actual mechanism behind this finding. However, by reviewing several sources, we believe that various dietary habits, geographic regions, ethnic differences, and changes in oestrogen concentration in women could have potential influences on cardiovascular outcomes. Our research might provide evidence of baseline concentrations of PUFAs on their clinical utility in the relation to the CVD risk, which may be a manifestation indicating the indirect outcome of dietary supplement habits that would allow proper dietary recommendations especially in Asian populations.

The metabolic enzymes, D5D and D6D, have been associated with many chronic diseases and inflammatory responses^38,39^. Hypothetically, a high enzymatic activity may indicate a peculiar susceptibility of the arterial wall to inflammatory stimuli during the atherosclerotic process^39^. Higher activities of D6D and lower activities of D5D are associated with the CHD risk^29^. In our study, we also noted a significant inverse dose-response trend of the D5D metabolic enzyme in Model 2. D5D-related processes are a key step in humans, and genetic variations in this step among individuals may regulate the efficiency of the conversion of high concentrations of long-chain PUFAs, such as AA^40^. Genes suppressing the activity of both D5D and D6D may lead to low plasma and tissue concentrations of PUFAs and their products^41^. The decrease in beneficial eicosanoids could increase inflammatory metabolites that could cause endothelial dysfunction and thus accelerate the progression of low-grade inflammation and atherosclerosis.

The biological mechanisms underlying the relationship of D5D activity and LA with the CVD risk are not well understood. However, the numerous biologically active compounds produced from PUFAs may play a role. In addition to facilitating inflammatory responses, LA and AA metabolites help reduce inflammation and promote resolution^25^. These anti-inflammatory metabolites could inhibit leukocyte activation and platelet aggregation and boost endothelial cell production of other anti-inflammatory metabolites^5,42^. D5D and D6D are the key enzymes that produce long-chain PUFAs, such as AA and EPA. The protective effect of higher D5D activities may be attributed to increased production of anti-inflammatory eicosanoids and lipoxins, resolvins, and protectins from AA, EPA, and DHA^39^. Without the D5D enzyme, dihomo-γ-linolenic acid cannot be metabolised to long-chain PUFAs that are essential precursors to numerous anti-inflammatory metabolites. Breakdown in the balance between anti- and pro-inflammatory metabolites would then be expected to increase the risk for some chronic diseases.

Our study has several strengths. First, it is a valuable report in Asia with a large sample size and a long-term follow-up period. In addition, the use of a community-based population could help reduce the possibility of selection bias. To control for potential confounding factors, this study also incorporated important socioeconomic and lifestyle factors into several models. We believe a report on the observation of a strong relationship in Asia or Taiwan could provide an important evidence of a potential preventive measure in reducing future CVD risks. Second, we had a clear disease ascertainment strategy. Lastly, we were able to measure fatty acid biomarkers and had good internal standards for measuring plasma fatty acids.

This study also had several potential limitations. First, because we only measured the concentrations of fatty acids once, our results might be attenuated by intra-individual variations. However, Wu *et al*. also measured plasma fatty acids once and used them as biomarkers from 1992 to 1993^43^. Their single measurement still exhibited that the within-person correlations for N-6 PUFAs from baseline to 13 years were moderate (0.49 for LA and 0.60 for AA), showing that the correlation of fatty acids is still high. Furthermore, the actual effect must be stronger because the effects of fatty acids could be underestimated. Second, food frequency questionnaires related to dietary fat intake and dietary supplement habits were not administered. However, we provided adequate data in presenting the associations between N-6 fatty acid concentrations and incident CVD risks, which may be a manifestation indicating the indirect outcome of dietary supplement habits. Lastly, the participants in this study were predominately ethnic Chinese. Our observations might not be generalisable to other ethnic groups.

## Conclusion

In this community-based cohort study in Taiwan, the total N-6 PUFA and LA concentrations were inversely associated with the risk of incident CVD in a dose-response manner. Further clinical trial studies are warranted to investigate the effect of N-6 fatty acid concentrations and dietary habits on cardiovascular health.

## Methods

### Study design and population

The design of this cohort study has previously been described^44,45^. Briefly, the study was conducted in Chin-Shan Township, Taiwan, and began in 1990; Interview questionnaires in 2-year cycles were administered for collecting data on anthropometry, lifestyle, and medical conditions. The study protocol was approved by the Institutional Review Board (IRB number: 201605105RINA) of the National Taiwan University Hospital. Formal informed consents were obtained for all participants for entering the study. Participants were aged over 35 years. All methods were performed in accordance with the relevant guidelines and regulations.

### Determination of fatty acid profiles using gas chromatography

Biochemical measurements (including fatty acid profiles) were performed once at baseline in 1835 participants. The procedures used for blood sample collection and fatty acid measurement have been previously reported^30,44^. Briefly, a total of 29 individual fatty acids were identified in this study. The relative quantity of each fatty acid (% of the total fatty acids) was determined by integrating the area beneath the peak and dividing the results by the total area of all fatty acids^30^. The fatty acid profiles included SFAs, trans fat, polyunsaturated fat, monounsaturated fat, DHA, EPA, and the activity of metabolic enzymes (D5D and D6D). The P/S ratio was defined as PUFAs: SFAs. The inter-assay coefficients of variation were 6.5%, 4.3%, 4.0%, and 7.5% for LA, AA, EPA, and DHA, respectively.

### Ascertainment of outcomes

The primary endpoints were the time to the first incident CVD events, including CHDs and stroke. Data on incident non-fatal ischaemic and stroke events were obtained from annually administered questionnaires. All diagnoses were confirmed by neurologists and internists. Incident CHD included cases of nonfatal myocardial infarction, angina, and hospitalisation due to percutaneous coronary intervention or coronary bypass surgery. Stroke events included cases of an incident neurological deficit of vascular origin lasting longer than 24 hours and those diagnosed via imaging studies in support of the evidence. The follow-up period ended when the subjects developed new-onset CVD before December 31, 2014, died before December 31, 2014, or lived beyond December 31, 2014.

### Statistical analysis

All participants were categorised into quartiles according to the blood concentrations of the fatty acid profiles and metabolic enzymes. Continuous variables were presented as means ± standard deviations. The difference among the quartiles was assessed using analysis of variance. The chi-square test was used to test the significance among the categorical data.

To compare the different fatty acids in predicting the CVD risk, we used the following strategies: Multivariable Cox proportional hazard models were used to estimate the HR and their respective 95% CI (see Supplementary Materials for the details of Models 1–3). All analyses were performed using SAS version 9.3 (SAS Institute, Cary, NC) and R version 3.3.0. Two-tailed *P*-values of <0.05 were considered to indicate statistical significance.

## Supplementary information

SUPPLEMENTARY INFO (material)

## References

[1] Libby P, Ridker PM, Maseri A (2002). Inflammation and Atherosclerosis. Circulation.

[2] Davignon J (1978). The lipid hypothesis. Arch Surg.

[3] Calder PC (2001). Polyunsaturated Fatty Acids, Inflammation, and Immunity. Lipids.

[4] Tapiero H, Ba GN, Couvreur P, Tew KD (2002). Polyunsaturated fatty acids (PUFA) and eicosanoids in human health and pathologies. Biomedicine & Pharmacotherapy.

[5] Calder PC (2006). Polyunsaturated fatty acids and inflammation. Prostaglandins, Leukotrienes and Essential Fatty Acids.

[6] Das UN (2007). A defect in the activity of D6 and D5 desaturases may be a factor in the initiation and progression of atherosclerosis. Prostaglandins, Leukotrienes and Essential Fatty Acids.

[7] Hu FB (1997). Dietary fat intake and the risk of coronary heart disease in women. The New England Journal of Medicine.

[8] Jakobsen MU (2009). Major types of dietary fat and risk of coronary heart disease: a pooled analysis of 11 cohort studies. American Journal of Clinical Nutrition.

[9] Jackson, R. L., Taunton, O. D., Morrisett, J. D. & Gotto, A. M. The Role of Dietary Polyunsaturated Fat in Lowering Blood Cholesterol in Man. *Circulation Research* **42** (1978). 10.1161/01.res.42.4.447 204426

[10] Temme EHM, Mensink RP, Hornstra G (1996). Comparison of the effects of diets enriched in lauric, palmitic, or oleic acids on serum lipids and lipoproteins in healthy women and men. American Society for Clinical Nutrition.

[11] Kris-Etherton PM, Yu S (1997). Individual fatty acid effects on plasma lipids and lipoproteins: human studies. American Journal of Clinical Nutrition.

[12] Zuliani G (2009). The role of polyunsaturated fatty acids (PUFA) in the treatment of dyslipidemias. Curr Pharm Des.

[13] Petersson H, Basu S, Cederholm T, Riserus U (2008). Serum fatty acid composition and indices of stearoyl-CoA desaturase activity are associated with systemic inflammation: longitudinal analyses in middle-aged men. British Journal of Nutrition.

[14] Jacobs S (2015). Evaluation of various biomarkers as potential mediators of the association between Delta5 desaturase, Delta6 desaturase, and stearoyl-CoA desaturase activity and incident type 2 diabetes in the European Prospective Investigation into Cancer and Nutrition-Potsdam Study. The American Journal of Clinical Nutrition.

[15] Kroger J, Schulze MB (2012). Recent insights into the relation of Delta5 desaturase and Delta6 desaturase activity to the development of type 2 diabetes. Current Opinion in Lipidology.

[16] Warensjö E, Sundström J, Vessby B, Cederholm T, Risérus U (2008). Markers of dietary fat quality and fatty acid desaturation as predictors of total and cardiovascular mortality: a population-based prospective study. The American Journal of Clinical Nutrition.

[17] Martinelli N (2008). FADS genotypes and desaturase activity estimated by the ratio of arachidonic acid to linoleic acid are associated with inflammation and coronary artery disease. The American Journal of Clinical Nutrition.

[18] Lu Y (2010). Dietary n-3 and n-6 polyunsaturated fatty acid intake interacts with FADS1 genetic variation to affect total and HDL-cholesterol concentrations in the Doetinchem Cohort Study. The American Journal of Clinical Nutrition.

[19] Do HJ, Chung HK, Moon J, Shin MJ (2011). Relationship between the estimates of desaturase activities and cardiometabolic phenotypes in Koreans. Journal of Clinical Biochemistry and Nutrition.

[20] Kim SR, Jeon SY, Lee S-M (2015). The association of cardiovascular risk factors with saturated fatty acids and fatty acid desaturase indices in erythrocyte in middle-aged Korean adults. Lipids in Health and Disease.

[21] Saini RK, Keum YS (2018). Omega-3 and omega-6 polyunsaturated fatty acids: Dietary sources, metabolism, and significance - A review. Life Sci.

[22] Harris WS (2009). Omega-6 fatty acids and risk for cardiovascular disease: a science advisory from the American Heart Association Nutrition Subcommittee of the Council on Nutrition, Physical Activity, and Metabolism; Council on Cardiovascular Nursing; and Council on Epidemiology and Prevention. Circulation.

[23] Mozaffarian D (2005). Interplay Between Different Polyunsaturated Fatty Acids and Risk of Coronary Heart Disease in Men. Circulation.

[24] Laaksonen, D. E., Nyyssönen, K., Niskanen, L., Rissanen, T. H. & Jukka T. S. Prediction of Cardiovascular Mortality in Middle-aged Men by Dietary and Serum Linoleic and Polyunsaturated Fatty Acids. *Archives of internal medicine* **165** (2005). 10.1001/archinte.165.2.193 15668366

[25] Fritsche KL (2008). Too much linoleic acid promotes inflammation-doesn’t it?. Prostaglandins, Leukotrienes & Essential Fatty Acids (PLEFA).

[26] Czernichow S, Thomas D, Bruckert E (2010). n-6 Fatty acids and cardiovascular health: a review of the evidence for dietary intake recommendations. British Journal of Nutrition.

[27] Farvid MS (2014). Dietary linoleic acid and risk of coronary heart disease: a systematic review and meta-analysis of prospective cohort studies. Circulation.

[28] Harris WS, Shearer GC (2014). Omega-6 fatty acids and cardiovascular disease: friend, not foe?. Circulation.

[29] Matthan N. R. *et al*. Plasma phospholipid fatty acid biomarkers of dietary fat quality and endogenous metabolism predict coronary heart disease risk: a nested case-control study within the Women’s Health Initiative observational study. *Journal of the American Heart Association* **3**, 10.1161/JAHA.113.000764 (2014). 10.1161/JAHA.113.000764 PMC4310362 25122663

[30] Chien KL (2013). Comparison of predictive performance of various fatty acids for the risk of cardiovascular disease events and all-cause deaths in a community-based cohort. Atherosclerosis.

[31] Mozaffarian D, Wu JH (2011). Omega-3 Fatty Acids and Cardiovascular Disease Effects on Risk Factors, Molecular Pathways, and Clinical Events. Journal of the American College of Cardiology.

[32] Bucher HC, Hengstler P, Schindler C, Meier G (2002). N-3 Polyunsaturated Fatty Acids in Coronary Heart Disease: A Meta-analysis of Randomized Controlled Trials. The American Journal of Medicine.

[33] Kwak SM, Myung SK, Lee YJ, Seo HG, Korean Meta-analysis Study, G. (2012). Efficacy of omega-3 fatty acid supplements (eicosapentaenoic acid and docosahexaenoic acid) in the secondary prevention of cardiovascular disease: a meta-analysis of randomized, double-blind, placebo-controlled trials. Archives of internal medicine.

[34] Harris William, Tintle Nathan, Ramachandran Vasan (2018). Erythrocyte n-6 Fatty Acids and Risk for Cardiovascular Outcomes and Total Mortality in the Framingham Heart Study. Nutrients.

[35] Marklund M (2019). Biomarkers of Dietary Omega-6 Fatty Acids and Incident Cardiovascular Disease and Mortality. Circulation.

[36] Menotti A, Kromhout D, Puddu PE (2017). Baseline fatty acids, food groups, a diet score and 50-year all-cause mortality rates. An ecological analysis of the Seven Countries Study. Ann Med.

[37] Kromhout D, Menotti A, Alberti-Fidanza A, Puddu PE (2018). Comparative ecologic relationships of saturated fat, sucrose, food groups, and a Mediterranean food pattern score to 50-year coronary heart disease mortality rates among 16 cohorts of the Seven Countries Study. Eur J Clin Nutr.

[38] Nakamura MT, Nara TY (2004). Structure, function, and dietary regulation of delta6, delta5, and delta9 desaturases. Annual Review of Nutrition.

[39] Tosi, F., Sartori, F., Guarini, P., Olivieri O. & Martinelli N. In *Oxidative Stress and Inflammation in Non-communicable Diseases - Molecular Mechanisms and Perspectives in Therapeutics* Vol. 824 61–81 (Springer International Publishing, 2014).

[40] Mathias RA (2010). FADS genetic variants and omega-6 polyunsaturated fatty acid metabolism in a homogeneous island population. The Journal of Lipid Research.

[41] Das Undurti N (2010). A defect in Δ6 and Δ5 desaturases may be a factor in the initiation and progression of insulin resistance, the metabolic syndrome and ischemic heart disease in South Asians. Lipids in Health and Disease.

[42] Das UN (2008). Can essential fatty acids reduce the burden of disease(s)?. Lipids in Health and Disease.

[43] Wu JH (2014). Circulating omega-6 polyunsaturated fatty acids and total and cause-specific mortality: the Cardiovascular Health Study. Circulation.

[44] Lee YT (2000). Chin-Shan Community Cardiovascular Cohort in Taiwan–baseline data and five-year follow-up morbidity and mortality. Journal of Clinical Epidemiology.

[45] Chien KL (2008). Plasma Uric Acid and the Risk of Type 2 Diabetes in a Chinese Community. Clin Chem.

