# Supplementary material for: Association between Plasma N-6 Polyunsaturated Fatty Acids Levels and the Risk of Cardiovascular Disease in a Community-based Cohort Study
Source: Sci Rep. 2019 Dec 17;9:19298. doi: 10.1038/s41598-019-55686-7 (PMC6917802; doi:10.1038/s41598-019-55686-7)
Supplement: Supplementary file 1 — SUPPLEMENTARY INFO (material) [file 41598_2019_55686_MOESM1_ESM.docx]

**Association between Plasma N-6 Polyunsaturated Fatty Acids Levels and the Risk of Cardiovascular Disease in a Community-based Cohort Study**

Wei-Sin Yang, Yun-Yu Chen, Pei-Chun Chen, Hsiu-Ching Hsu, Ta-Chen Su, Hung-Ju Lin, Ming-Fong Chen, Yuan-Teh Lee, Kuo-Liong Chien

**Supplementary Information**

**Methods**

**Study design and study population**

Blood specimens were collected at three time points during 1992–2000 (1992–1993, 1994–1995, and 1999–2000; Fig. 1). The validity and reproducibility of the collected data and measurements have also previously been reported in detail.[^1^](#_ENREF_1)

**Statistical analysis**

To examine the correlation between fatty acids and the risk factors of cardiovascular diseases (CVD), Spearman correlation coefficients (r) were adjusted for age and gender.

To compare different fatty acids in predicting CVD risk, we used the following strategies:

The incidence rates of CVD events were calculated by dividing the number of cases by the number of person-years of follow-up for each quartile group. To test for linear trends across categories of lipid markers, we selected median fatty acid levels within quartiles as a continuous variable.

Multivariable Cox proportional hazard models were used to estimate the hazard ratios and respective 95% confidence intervals: Model 1 was adjusted for age (35–44, 45–54, 55–64, 65–74, >=75 years old) and gender only; Model 2 included additional confounding factors: body mass index (<18, 18–20.9, 21–22.9, 23–24.9, >=25 kg/m^2^), smoking (yes/no or abstinence), current alcohol consumption habits (regular/none), marital status (single, married and living with spouse, separated, or divorced), education level (less than 9 years, at least 9 years), occupational status (not employed, manual labor, or office job), and regular exercise (yes/no); Model 3 included additional clinical variables: baseline hypertension (yes/no), diabetes (yes/no), continuous high-density lipoprotein and low-density lipoprotein cholesterol values.

To compare the effects between two biomarkers of fatty acids, a two-marker method was used to adjust for two biomarkers of fatty acids at the same time in Model 3. In addition, we also evaluated the joint effects of linoleic acid (LA) and n-3 polyunsaturated fatty acids (PUFAs) using a stratified analysis of joint effects based on Model 3 (we defined the lowest concentration of both as reference values).

We compared the performance of the models with and without fatty acid measurements using the area under the receiver operating characteristic (ROC) curve (AUC). Youden’s index was used to find various cut-off values of a positive diagnostic test result.[^2^](#_ENREF_2) Statistical differences in the AUCs were compared using the DeLong’s method to compare the discriminatory capability among the models. We also provided net reclassification improvement (NRI) for the comparison of nested models with and without fatty acids.[^3^](#_ENREF_3) A priori risk categories were defined according to the a priori risk categories of cardiovascular diseases (CVD) in NRI analysis (<15%, 15–20%, and ≥ 20%). The integrated discrimination improvement (IDI) is considered to be the difference between improvement in average sensitivity and any potential increase in average ‘one minus specificity’.[^3^](#_ENREF_3) We used the R package “PredictABEL” for the assessment of risk prediction models.[^4^](#_ENREF_4)

Potential non-linear associations were assessed semi-parametrically using restricted cubic splines. Multivariable hazard ratios of plasma fatty acid profiles were evaluated in patients with risk of CVD events by using restricted cubic splines from Cox models. The restricted cubic spline model was defined with four knots of plasma fatty acid level and was constructed using the R package “rms” for the assessment of risk prediction models.[^5^](#_ENREF_5)

We estimated the population attributable risk (PAR) in order to quantify the public health impact of an exposure on an outcome by using the R package “AF”.[^6^](#_ENREF_6) For time-to-event outcomes in cohort studies, the confounder-adjusted AF function was estimated using Cox proportional hazard regression based on Model 3. Furthermore, we defined plasma fatty acid concentrations lower than median value as the exposure and D6D higher than median value as the exposure. For the “times,” argument specifies the time points at which the attributable risk is supposed to be estimated. We have estimated the attributable risk at the median follow-up of 16 years.

**Results**

In Supplementary Table S1, linoleic acid (LA) was positively associated with other N-6 polyunsaturated fatty acids (N-6 PUFAs: arachidonic acid [AA] and gamma-linolenic acid [GLA]), N-3 PUFAs (alpha linolenic acid [ALA] and docosahexaenoic acid [DHA]), and P/S ratio. Furthermore, LA was also positively associated with delta-5 desaturase (D5D) metabolic enzyme (r = 0.35, *P*<0.001). LA was inversely associated with delta-6 desaturase (D6D) metabolic enzyme (r =–0.44, *P*<0.001) and other covariates like body mass index (BMI), low-density lipoprotein (LDL), systolic blood pressure (SBP), diastolic blood pressure (DBP), and triglyceride (TG) (r = –0.07 to –0.11).

When participants were stratified based on their joint LA and N-3 PUFA concentrations, those with the highest circulating levels of both LA and N-3 had 45% lower risk of incident CVD (adjusted HR=0.55; 95% CI, 0.34–0.89) relative to those with the lowest levels of both (adjusted HR=0.59; 95% CI, 0.34–1.02; Supplementary Figure S1). Supplementary Figure S2 showed the multivariable HRs of plasma phospholipid LA for individuals with risk of CVD events and little evidence for nonlinearity of hazard evaluated by restricted cubic splines (*P* for nonlinearity=0.84; *P* for trend=0.034).

**Supplementary References**

1 Lee Y.T., L. R. S., Sung F.C., Yang C.Y., Chien K.L., Chen W.J., Su T.C., Hsu H.C., Huang Y.C. Chin-Shan Community Cardiovascular Cohort in Taiwan–baseline data and five-year follow-up morbidity and mortality. *Journal of Clinical Epidemiology* **53**, 838-846 (2000).

2 Hanley J.A., M. B. J. A Method of Comparing under Receiver Operating Characteristic Curves the Same Cases. *Radiology* **148**, 839-843 (1983).

3 Pencina, M. J., D'Agostino, R. B., Sr., D'Agostino, R. B., Jr. & Vasan, R. S. Evaluating the added predictive ability of a new marker: from area under the ROC curve to reclassification and beyond. *Stat Med* **27**, 157-172; discussion 207-112, doi:10.1002/sim.2929 (2008).

4 Kundu, S., Aulchenko, Y. S., van Duijn, C. M. & Janssens, A. C. PredictABEL: an R package for the assessment of risk prediction models. *European Journal of Epidemiology* **26**, 261-264, doi:10.1007/s10654-011-9567-4 (2011).

5 rms: S functions for biostatistical/epidemiologic modeling, testing, estimation, validation, graphics, and prediction. (2009).

6 Dahlqwist E., Z. J., Pawitan Y., Sjölander A. Model-based estimation of the attributable fraction for cross-sectional, case–control and cohort studies using the R package AF. *Eur J Epidemiol*, doi:10.1007/s10654-016-0137-7 (2016).

**Supplementary Figure S1.**

Multivariable hazard ratios of plasma linoleic acid with risk of CVD, evaluated by restricted cubic splines from Cox models.

CVD: cardiovascular diseases; DHA: docosahexaenoic acid; EPA: eicosapentaenoic acid; FAs: atty acids; LA: linoleic acid; Ref: reference.


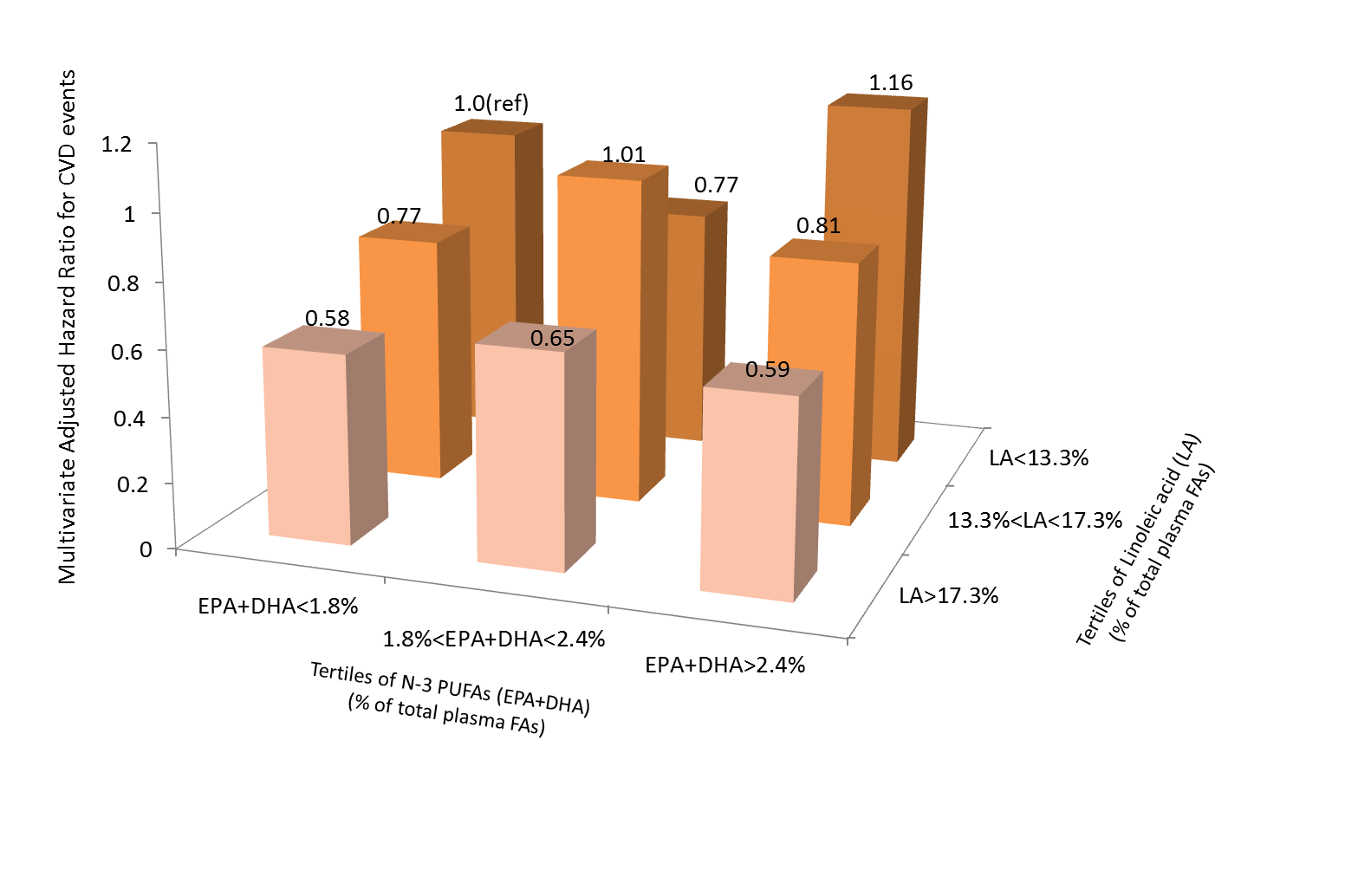


**Supplementary Figure S2.**

Smooth effect CVD risk of LA via restricted cubic model.

CVD: cardiovascular diseases; LA: linoleic acid; Ref: reference.


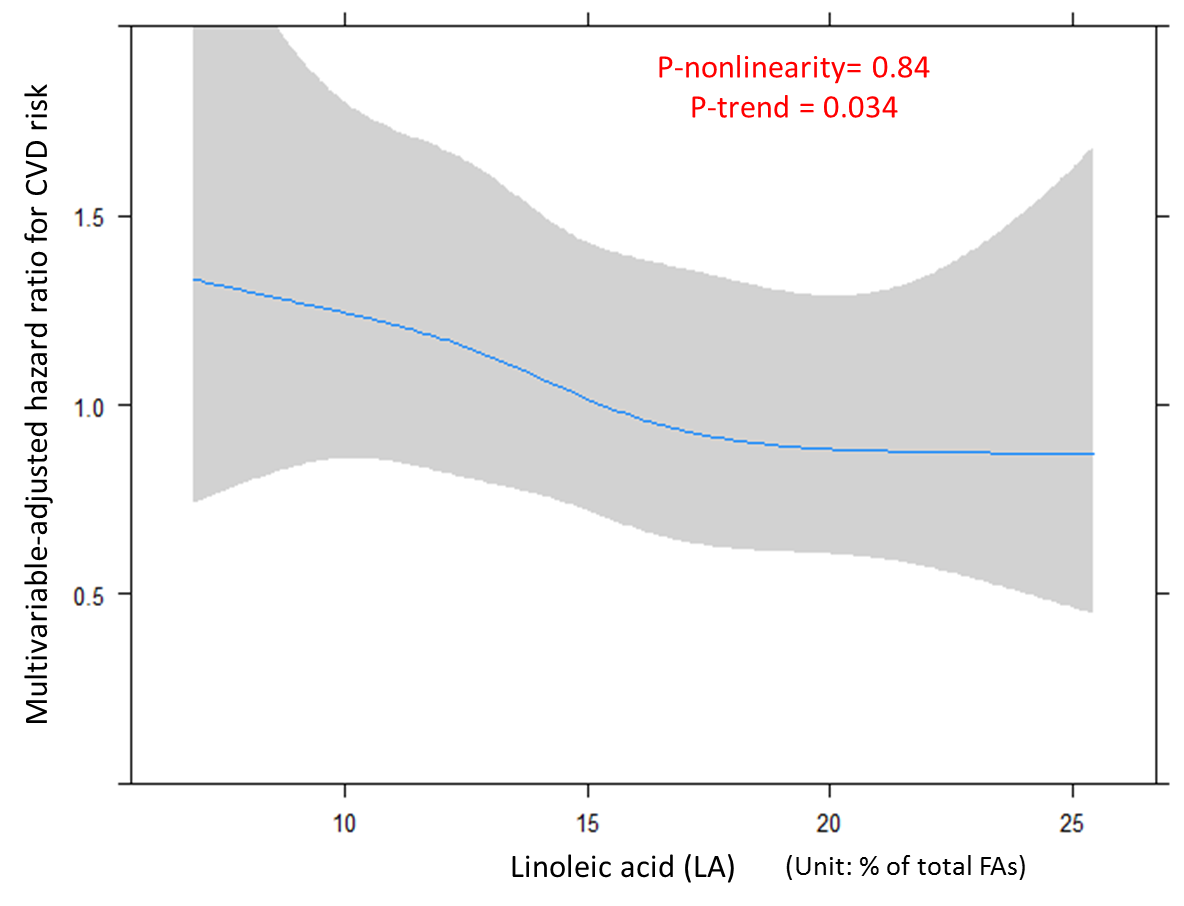


**Supplementary Table S1. Age and sex adjusted spearman correlations.**

|  | LA | GLA | AA | ALA | EPA | DHA | D5D | D6D | BMI | LDL | HDL | SBP | DBP | TG | P/S |
| --- | --- | --- | --- | --- | --- | --- | --- | --- | --- | --- | --- | --- | --- | --- | --- |
| LA | 1 | 0.09 | 0.43 | 0.43 | 0.06 | 0.28 | 0.35 | -0.44 | -0.09 | -0.07 | 0.07 | -0.09 | -0.09 | -0.11 | 0.90 |
| GLA |  | 1 | 0.16 | 0.19 | 0.02 | 0.02 | -0.11 | 0.83 | 0.13 | -0.06 | -0.01 | -0.01 | 0.05 | 0.03 | 0.13 |
| AA |  |  | 1 | -0.06 | 0.38 | 0.54 | 0.68 | -0.09 | -0.12 | -0.05 | 0.32 | -0.13 | -0.12 | -0.24 | 0.57 |
| ALA |  |  |  | 1 | -0.03 | 0.03 | -0.08 | -0.06 | 0.07 | -0.01 | -0.22 | 0.04 | 0.05 | 0.17 | 0.37 |
| EPA |  |  |  |  | 1 | 0.57 | 0.40 | -0.01 | -0.02 | -0.03 | 0.15 | 0.04 | 0.02 | -0.15 | 0.14 |
| DHA |  |  |  |  |  | 1 | 0.48 | -0.13 | -0.07 | 0.01 | 0.18 | -0.02 | -0.03 | -0.09 | 0.40 |
| D5D |  |  |  |  |  |  | 1 | -0.27 | -0.23 | -0.05 | 0.28 | -0.16 | -0.15 | -0.23 | 0.39 |
| D6D |  |  |  |  |  |  |  | 1 | 0.16 | -0.01 | -0.05 | 0.05 | 0.09 | 0.09 | -0.36 |
| BMI |  |  |  |  |  |  |  |  | 1 | 0.17 | -0.36 | 0.26 | 0.25 | 0.26 | -0.11 |
| LDL |  |  |  |  |  |  |  |  |  | 1 | -0.16 | 0.09 | 0.07 | 0.37 | -0.09 |
| HDL |  |  |  |  |  |  |  |  |  |  | 1 | -0.11 | -0.08 | -0.46 | 0.15 |
| SBP |  |  |  |  |  |  |  |  |  |  |  | 1 | 0.71 | 0.17 | -0.11 |
| DBP |  |  |  |  |  |  |  |  |  |  |  |  | 1 | 0.16 | -0.12 |
| TG |  |  |  |  |  |  |  |  |  |  |  |  |  | 1 | -0.18 |
| P/S |  |  |  |  |  |  |  |  |  |  |  |  |  |  | 1 |

AA: arachidonic acid; ALA: alpha linolenic acid; BMI: body mass index; BP: blood pressure; CAD: coronary artery diseases; D5D: delta-5 desaturase; D6D: delta-6 desaturase; DBP: diastolic blood pressure; DHA: docosahexaenoic acid; EPA: eicosapentaenoic acid; FAs: fatty acids; GLA: gamma-linolenic acid; HDL: high-density lipoprotein; LA: linoleic acid; LDL: low-density lipoprotein; PUFAs: polyunsaturated fatty acids; SBP: systolic blood pressure; TG: triglycerides.

P/S ratio was defined as PUFAs：saturated fatty acids (SFA).

**Supplementary Table S2. Hazard ratio of incidence CVD according to N-3 PUFAs**

| **N-3 PUFAs** | Q1 | Q2 | 95% CI | | | | | Q3 | 95% CI | | | | | Q4 | 95% CI | | | | | P-trend |
| --- | --- | --- | --- | --- | --- | --- | --- | --- | --- | --- | --- | --- | --- | --- | --- | --- | --- | --- | --- | --- |
| **Median** | 2.63 | 3.18 |  |  |  |  |  | 3.71 |  |  |  |  |  | 4.65 |  |  |  |  |  |  |
| **Case (n)** | 121 | 101 |  |  |  |  |  | 102 |  |  |  |  |  | 100 |  |  |  |  |  |  |
| **person-year** | 6205.6 | 7388.4 |  |  |  |  |  | 7698.1 |  |  |  |  |  | 7592.9 |  |  |  |  |  |  |
| **Rates/1000 py** | 19.5 | 13.7 |  |  |  |  |  | 13.3 |  |  |  |  |  | 13.2 |  |  |  |  |  |  |
| **Model 1** | 1 | 0.78 | ( | 0.60 | , | 1.02 | ) | 0.70 | ( | 0.53 | , | 0.92 | ) | 0.80 | ( | 0.6 | , | 1.05 | ) | 0.13 |
| **Model 2** | 1 | 0.85 | ( | 0.64 | , | 1.11 | ) | 0.75 | ( | 0.57 | , | 0.99 | ) | 0.85 | ( | 0.64 | , | 1.12 | ) | 0.25 |
| **Model 3** | 1 | 0.88 | ( | 0.66 | , | 1.17 | ) | 0.74 | ( | 0.55 | , | 0.98 | ) | 0.81 | ( | 0.60 | , | 1.09 | ) | 0.12 |
| **ALA** | Q1 | Q2 | 95% CI | | | | | Q3 | 95% CI | | | | | Q4 | 95% CI | | | | | p-trend |
| **Median** | 0.23 | 0.3 |  |  |  |  |  | 0.38 |  |  |  |  |  | 0.54 |  |  |  |  |  |  |
| **Case (n)** | 32 | 71 |  |  |  |  |  | 119 |  |  |  |  |  | 202 |  |  |  |  |  |  |
| **Person-years** | 1936.5 | 4568.9 |  |  |  |  |  | 8443.3 |  |  |  |  |  | 13999 |  |  |  |  |  |  |
| **Rates/1000 py** | 16.5 | 15.5 |  |  |  |  |  | 14.1 |  |  |  |  |  | 14.4 |  |  |  |  |  |  |
| **Model 1** | 1 | 0.86 | ( | 0.56 | , | 1.32 | ) | 0.83 | ( | 0.56 | , | 1.22 | ) | 0.91 | ( | 0.63 | , | 1.33 | ) | 0.80 |
| **Model 2** | 1 | 0.84 | ( | 0.55 | , | 1.30 | ) | 0.79 | ( | 0.53 | , | 1.17 | ) | 0.89 | ( | 0.61 | , | 1.29 | ) | 0.85 |
| **Model 3** | 1 | 0.77 | ( | 0.50 | , | 1.18 | ) | 0.71 | ( | 0.47 | , | 1.05 | ) | 0.73 | ( | 0.50 | , | 1.08 | ) | 0.39 |
| **EPA** | Q1 | Q2 | 95% CI | | | | | Q3 | 95% CI | | | | | Q4 | 95% CI | | | | | p-trend |
| **Median** | 0.21 | 0.3 |  |  |  |  |  | 0.38 |  |  |  |  |  | 0.56 |  |  |  |  |  |  |
| **Case (n)** | 112 | 97 |  |  |  |  |  | 100 |  |  |  |  |  | 115 |  |  |  |  |  |  |
| **Person-years** | 6773.9 | 7278.9 |  |  |  |  |  | 7724.3 |  |  |  |  |  | 7170.4 |  |  |  |  |  |  |
| **Rates/1000 py** | 16.5 | 13.3 |  |  |  |  |  | 12.9 |  |  |  |  |  | 16 |  |  |  |  |  |  |
| **Model 1** | 1 | 0.82 | ( | 0.62 | , | 1.09 | ) | 0.79 | ( | 0.59 | , | 1.04 | ) | 0.97 | ( | 0.74 | , | 1.28 | ) | 0.86 |
| **Model 2** | 1 | 0.78 | ( | 0.59 | , | 1.04 | ) | 0.77 | ( | 0.58 | , | 1.03 | ) | 0.97 | ( | 0.74 | , | 1.28 | ) | 0.76 |
| **Model 3** | 1 | 0.84 | ( | 0.63 | , | 1.13 | ) | 0.72 | ( | 0.54 | , | 0.97 | ) | 0.98 | ( | 0.74 | , | 1.3 | ) | 0.87 |
| **DHA** | Q1 | Q2 | 95% CI | | | | | Q3 | 95% CI | | | | | Q4 | 95% CI | | | | | p-trend |
| **Median** | 1.1 | 1.53 |  |  |  |  |  | 1.92 |  |  |  |  |  | 2.51 |  |  |  |  |  |  |
| **Case (n)** | 104 | 121 |  |  |  |  |  | 91 |  |  |  |  |  | 108 |  |  |  |  |  |  |
| **Person-years** | 6424.2 | 7113.6 |  |  |  |  |  | 7808.4 |  |  |  |  |  | 7601.4 |  |  |  |  |  |  |
| **Rates/1000 py** | 16.2 | 17 |  |  |  |  |  | 11.7 |  |  |  |  |  | 14.2 |  |  |  |  |  |  |
| **Model 1** | 1 | 1.17 | ( | 0.89 | , | 1.53 | ) | 0.76 | ( | 0.56 | , | 1.02 | ) | 1.03 | ( | 0.78 | , | 1.37 | ) | 0.58 |
| **Model 2** | 1 | 1.24 | ( | 0.94 | , | 1.64 | ) | 0.82 | ( | 0.60 | , | 1.10 | ) | 1.1 | ( | 0.82 | , | 1.48 | ) | 0.88 |
| **Model 3** | 1 | 1.17 | ( | 0.88 | , | 1.56 | ) | 0.8 | ( | 0.59 | , | 1.09 | ) | 1.04 | ( | 0.77 | , | 1.41 | ) | 0.64 |

Model 1: age, gender;

Model 2: Model 1+ body mass index, smoking, alcohol consumption habits, marital status, education level, occupation, and regular exercise;

Model 3: Model 2+ baseline hypertension, diabetes, continuous high-density lipoprotein and low-density lipoprotein cholesterol values.

ALA: alpha linolenic acid; CI: confidence interval; CVD: cardiovascular diseases; DHA: docosahexaenoic acid; EPA: eicosapentaenoic acid; PUFAs: polyunsaturated fatty acids; py: person-years.

**Supplementary Table S3. Hazard ratio of incidence cardiovascular diseases according to P/S Ratio.**

| P/S Ratio | Q1 | Q2 |  |  | Q3 |  |  | Q4 |  |  | p-trend |
| --- | --- | --- | --- | --- | --- | --- | --- | --- | --- | --- | --- |
| Median | 0.43 | 0.53 |  |  | 0.62 |  |  | 0.77 |  |  |  |
| Case (n) | 126 | 129 |  |  | 88 |  |  | 80 |  |  |  |
| Person-years (py) | 6577.4 | 6541.7 |  |  | 7394.1 |  |  | 8236.8 |  |  |  |
| Rates/1000 py | 19.2 | 19.7 |  |  | 11.9 |  |  | 9.7 |  |  |  |
| Model 1 | 1 | 1.04 | 0.81 | 1.34 | 0.64 | 0.49 | 0.85 | 0.57 | 0.42 | 0.76 | <0.001 |
| Model 2 | 1 | 1.09 | 0.84 | 1.40 | 0.66 | 0.50 | 0.87 | 0.58 | 0.43 | 0.79 | <0.001 |
| Model 3 | 1 | 1.18 | 0.91 | 1.53 | 0.70 | 0.53 | 0.93 | 0.58 | 0.42 | 0.80 | <0.001 |

Model 1: age, gender;

Model 2: Model 1+ body mass index, smoking, alcohol consumption habits, marital status, education level, occupation, and regular exercise;

Model 3: Model 2+ baseline hypertension, diabetes, continuous high-density lipoprotein and low-density lipoprotein cholesterol values.

**Supplementary Table S4. Stratified effects of N-6 PUFAs by different genders in various models.**

| Gender | Models | Hazard ratio (95% confidence interval) | | | | P trend |
| --- | --- | --- | --- | --- | --- | --- |
|  |  | Q1 | Q2 | Q3 | Q4 |  |
| Men | Median (n) | 20.8  (n=265) | 24.7  (n=272) | 27.2  (n=246) | 30.9  (n=236) |  |
|  | Model 0 | 1 (reference) | 0.71 (0.51, 0.99) | 0.68 (0.49, 0.96) | 0.48 (0.33, 0.69) | <0.001 |
|  | Model 1 | 1 (reference) | 0.72 (0.51, 1.00) | 0.73 (0.52, 1.02) | 0.58 (0.40, 0.83) | 0.003 |
|  | Model 2 | 1 (reference) | 0.72 (0.51, 1.01) | 0.75 (0.53, 1.05) | 0.57 (0.39, 0.83) | 0.003 |
|  | Model 3 | 1 (reference) | 0.73 (0.52, 1.02) | 0.83 (0.58, 1.18) | 0.58 (0.39, 0.86) | 0.01 |
| Women | Median (n) | 21.3  (n=194) | 24.5  (n=188) | 27.2  (n=211) | 31.7  (n=222) |  |
|  | Model 0 | 1 (reference) | 0.95 (0.65, 1.39) | 0.52 (0.34, 0.80) | 0.44 (0.29, 0.67) | <0.001 |
|  | Model 1 | 1 (reference) | 1.01 (0.69, 1.48) | 0.57 (0.38, 0.87) | 0.53 (0.34, 0.82) | 0.001 |
|  | Model 2 | 1 (reference) | 1.02 (0.69, 1.50) | 0.58 (0.38, 0.89) | 0.54 (0.35, 0.84) | 0.001 |
|  | Model 3 | 1 (reference) | 1.12 (0.75, 1.67) | 0.62 (0.40, 0.95) | 0.61 (0.39, 0.98) | 0.006 |

We tested if variables of gender and N-6 PUFAs have statistical interaction effect, and found that a significantly statistical interaction of N-6 PUFAs*gender (interaction P <0.001) and quartiles of N-6 PUFAs*gender (interaction P <0.001) in Model 0.

Model 0: crude effect;

Model 1: age;

Model 2: Model 1+ body mass index, smoking, alcohol consumption habits, marital status, education level, occupation, and regular exercise;

Model 3: Model 2+ baseline hypertension, diabetes, continuous high-density lipoprotein and low-density lipoprotein cholesterol values.

PUFAs: polyunsaturated fatty acids.

**Supplementary Table S5. Combination of two fatty acids adjusted confounding factor in multivariate adjusted models**

| Model |  |  | **HR (95% CI)** | | | | | **P trend** |  |  | **HR (95% CI)** | | | | | **P trend** |
| --- | --- | --- | --- | --- | --- | --- | --- | --- | --- | --- | --- | --- | --- | --- | --- | --- |
|  |  |  |  |  |  |  |  |  |  |  |  |  |  |  |  |  |
| 1 | LA | 0.6 | ( | 0.38 | , | 0.93 | ) | 0.019 | N-3 PUFAs (EPA+DHA) | 1.02 | ( | 0.67 | , | 1.53 | ) | 0.94 |
| 2 | LA | 0.59 | ( | 0.38 | , | 0.92 | ) | 0.020 | D5D | 0.98 | ( | 0.64 | , | 1.50 | ) | 0.91 |
| 3 | D5D | 0.84 | ( | 0.55 | , | 1.28 | ) | 0.67 | D6D | 0.93 | ( | 0.62 | , | 1.39 | ) | 0.52 |
| 4 | D5D | 0.88 | ( | 0.60 | , | 1.37 | ) | 0.67 | N-3 PUFA (EPA+DHA) | 0.95 | ( | 0.62 | , | 1.47 | ) | 0.75 |

Results are from separate models of each combination of fatty acid profiles. HR and 95% CI in the highest quartile was compared with the lowest quartile of each fatty acid. P for trend across quartiles of each profile, and each fatty acid profile was added to the multivariate adjusted model (Model 3) in quartile with 3 dummy variables.

CI: confidence interval; D5D: delta-5 desaturase; D6D: delta-6 desaturase; DHA: docosahexaenoic acid; EPA: eicosapentaenoic acid; HR: hazard ratio; LA: linoleic acid; PUFAs: polyunsaturated fatty acids.

**Supplementary Table S6. Net reclassification tables of the models with and without n-6 PUFAs concentrations in the model.**

|  |  |  |  |  |
| --- | --- | --- | --- | --- |
| Controls | Low | Moderate | High | Total |
| Low | 8 | 5 | 0 | 13 |
| Moderate | 34 | 52 | 37 | 123 |
| High | 0 | 63 | 360 | 423 |
| Total | 42 | 120 | 397 | 559 |
|  |  |  |  |  |
| Cases | Low | Moderate | High | Total |
| Low | 1 | 0 | 0 | 1 |
| Moderate | 6 | 5 | 16 | 27 |
| High | 0 | 17 | 191 | 208 |
| Total | 7 | 22 | 207 | 236 |

Defined categories: <15% (low), 15-20% (moderate), >20% (high).

PUFAs: polyunsaturated fatty acids.

**Supplementary Table S7. Performance measures of the models including various fatty acid levels**

| **Quartile fatty acids** | **NRI%** |  | **95%CI** | | |  | **P-value** | **IDI%** | **95%CI** | | | | | **P-value** |
| --- | --- | --- | --- | --- | --- | --- | --- | --- | --- | --- | --- | --- | --- | --- |
| Base model |  |  |  |  |  |  |  |  |  |  |  |  |  |  |
| + N-6 PUFAs | 7.20 | ( | 0.64 | , | 13.7 | ) | 0.032 | 0.79 | ( | 0.16 | , | 1.43 | ) | 0.014 |
| + N-3 PUFAs (EPA+DHA) | 4.22 | ( | -0.35 | , | 8.79 | ) | 0.07 | 0.45 | ( | -0.02 | , | 0.92 | ) | 0.06 |
| + D5D | 0.27 | ( | -3.72 | , | 4.27 | ) | 0.89 | 0.17 | ( | -0.12 | , | 0.46 | ) | 0.26 |
| + D6D | 5.12 | ( | -0.28 | , | 10.5 | ) | 0.06 | 0.49 | ( | -0.05 | , | 1.03 | ) | 0.07 |

NRI cutoff points: <5%, 5%-10%, 10%-20%, >20%;

CI: confidence interval; D5D: delta-5 desaturase; D6D: delta-6 desaturase; DHA: docosahexaenoic acid; EPA: eicosapentaenoic acid; IDI: integrated discrimination improvement; NRI: net reclassification improvement; PUFAs: polyunsaturated fatty acids.

**Supplementary Table S8. Estimate of the population attributable risk of PUFAs and metabolic enzymes.**

|  | Cut-off point (median) | PAR% | 95% CI | P-value |
| --- | --- | --- | --- | --- |
| n-6 PUFAs | <26% as exposure | 20.7 | (8.2 - 33.2) | 0.001 |
| LA | <15% as exposure | 10.8 | (-1.9 -23.4) | 0.10 |
| n-3 PUFAs | <2% as exposure | 0.5 | (-11.4 - 12.4) | 0.93 |
| D5D | <4 as exposure | -0.08 | (-13.4 - 13.2) | 0.99 |
| D6D | >=0.01 as exposure | 5.8 | (-5.6 - 17.2) | 0.32 |

* Specifies the time point at median follow-up years: 16 years.

* Values represent percent of fatty acids, except for D5D and D6D.

* Adjusted (Model 3): age, gender, body mass index, smoking, alcohol consumption habits, marital status, education level, occupation, and regular exercise, baseline hypertension, diabetes, continuous high-density lipoprotein and low-density lipoprotein cholesterol values.

CI: confidence interval; D5D: delta-5 desaturase; D6D: delta-6 desaturase; DHA: docosahexaenoic acid; LA: linoleic acid; PAR: population attributable risk; PUFAs: polyunsaturated fatty acids.
